# Supplementary material for: Taxonomic analysis of Paraguayan samples of Homonota fasciata Duméril & Bibron (1836) with the revalidation of Homonota horrida Burmeister (1861) (Reptilia: Squamata: Phyllodactylidae) and the description of a new species
Source: PeerJ. 2017 Aug 8;5:e3523. doi: 10.7717/peerj.3523 (PMC5553347; doi:10.7717/peerj.3523)
Supplement: Supplemental Information 1 — Supplementary information (Appendix S1–S6) [file peerj-05-3523-s001.pdf]

Taxonomic analysis of Paraguayan samples of *Homonota fasciata* Duméril & Bibron (1836) with the revalidation of *Homonota horrida* Burmeister (1861) (Reptilia: Squamata: Phyllodactylidae) and the description of a new species

Cacciali P, Morando M, Medina CD, Köhler G, Motte M, Avila LJ - PeerJ 5:e3523; DOI 10.7717/peerj.3523

Supplementary Information

Appendix S1. GenBank accession numbers (16S)........ 2

Appendix S2. Localities of specimens used for genetic analyses........ 3

Appendix S3. GenBank accession numbers (multi-genes)........ 6

Appendix S4. Primers used for genetic analyses........ 9

Appendix S5. Examined specimens for morphology........ 12

Appendix S6. Results of ABGD analysis........ 15

Appendix S7. Results of PTP analysis........ 17

Appendix S8. Individual gene trees........ 18

Appendix S9. Concatenated trees........ 24

Appendix S10. Most contributing variables for DA........ 27

Appendix S11. Matrix of metric data........ 28

Appendix S12. Matrix of meristic data........ 31

Appendix S1

GenBank accession numbers (16S)

GenBank accession numbers (GBAN) of specimens used for genetic analyses with 16S mitochondrial gene for each voucher (institutional acronyms follow Sabaj Pérez, 2014). Specimen indicated with [1] is currently BYU 47941.

Country	Specimen	GBAN
Argentina	LJAMM-CNP 5047[1]	MF278828
	LJAMM-CNP 10495	MF278829
	LJAMM-CNP 10576	MF278830
Paraguay	MNHNP 11873	MF278831
	MNHNP 12238	MF278832
	SMF 101984	MF278833

Appendix S2

Localities of specimens used for genetic analyses

Codes for countries are: ARG (Argentina), BRA (Brazil), CHI (Chile), PAR (Paraguay), and PER (Peru). Locality information is provided from the major political division of the country to the most specific location data. References to routes are RP (Provincial Route) and RN (National Route). Elevation is given in meters above sea level. Coordinates given with only one decimal are approximately, in which case elevation is not provided. Species treated as "*Homonota* sp. A" and "*Homonota* sp. B" were formerly referred as *H. fasciata*. Specimens with superindexes ([1–3]) are currently in BYU: 1- BYU 47960, 2- BYU 47941, 3- 47931.

Species	Voucher	Country	Specific locality	Lat/Long	Elevation
H. andicola	LJAMM-CNP 12490	ARG	Mendoza, Las Heras, RP 39, 3 km S from limit San Juan-Mendoza	-32.098º, -69.371º	2231
	LJAMM-CNP 12493	ARG	Mendoza, Las Heras, RP 39, 3 km S from limit San Juan-Mendoza	-32.098º, -69.371º	2231
	LJAMM-CNP 12494	ARG	Mendoza, Las Heras, RP 39, 3 km S from limit San Juan-Mendoza	-32.098º, -69.371º	2231
	LJAMM-CNP 12495	ARG	Mendoza, Las Heras, RP 39, 3 km S from limit San Juan-Mendoza	-32.098º, -69.371º	2231
H. borellii	LJAMM-CNP 5842[1]	ARG	La Rioja, Anillaco	-28.814º, -66.933º	1362
	LJAMM-CNP 12116	ARG	Santiago del Estero, Ojo de Agua, Sierra de Ambargasta	-29.249º, -63.920º	314
	LJAMM-CNP 12125	ARG	Santiago del Estero, Ojo de Agua, Sierra de Ambargasta	-29.249º, -63.920º	314
H. darwinii	LJAMM-CNP 9266	ARG	Santa Cruz, RP 45, E of Estancia El Cerrito	-46.263º, -71.378º	640
	LJAMM-CNP 9813	ARG	Santa Cruz, 4 km NE of Puerto Deseado	-47.715º, -65.839º	16
	LJAMM-CNP 10638	ARG	Santa Cruz, RP 77, ~500 m from detour to Estancia La María	-48.498º, -68.864º	214
	LJAMM-CNP 11424	ARG	Santa Cruz, RN 3, 5 km N of Estancia El Rancho	-46.781º, -67.354º	266
	LJAMM-CNP 11432	ARG	Santa Cruz, NR 3, 12 km S of detour to Cerro Vanguardia	-48.512º, -67.731º	85
Homonota sp. A	LJAMM-CNP 5047[2]	ARG	Mendoza, RP 190, 2 km S (on road) from Punta del Agua	-35.538º, -68.080º	795
	LJAMM-CNP 10495	ARG	Mendoza, RP 190, 2 km S (on road) from Punta del Agua	-35.538º, -68.080º	795
	LJAMM-CNP 10505	ARG	La Pampa, RP 27, 4 km N of intersection with RP 14	-36.668º, -68.022º	767

Species	Voucher	Country	Specific locality	Lat/Long	Elevation
	LJAMM-CNP 10528	ARG	La Pampa, RP 16, 23 km W of intersection with RN 151	-37.075º, -67.785º	538
	LJAMM-CNP 10576	ARG	Mendoza, Secondary road 88 m from RP 190, 2.3 km S (on road) from Punta del Agua	-35.541º, -68.079º	786
	LJAMM-CNP 10577	ARG	Mendoza, Secondary road 88 m from RP 190, 2.3 km S (on road) from Punta del Agua	-35.541º, -68.079º	786
	LJAMM-CNP 12415	ARG	Neuquén, Secondary road, 80 m from RP 7, 13 km NW of Añelo	-38.264º, -68.891º	477
Homonota sp. B	MNHNP 11406	PAR	Boquerón, Parque Nacional Teniente Enciso	-21.209º, -61.655º	253
	MNHNP 11409	PAR	Boquerón, Parque Nacional Teniente Enciso	-21.209º, -61.655º	253
	MNHNP 11873	PAR	Boquerón, Cruce San Miguel, in front to Parque Nacional Teniente Enciso	-21.203º, -61.662º	254
	MNHNP 12238	PAR	Boquerón, Fortín Mayor Infante Rivarola	-21.679º, -62.401º	277
	SMF 101984	PAR	Boquerón, Fortín Mayor Infante Rivarola	-21.679º, -62.401º	277
H. rupicola	RUPI-1	PAR	Cordillera, Cerro Pedregal	-25.515º, -57.042º	288
	RUPI-2	PAR	Cordillera, Cerro Pedregal	-25.515º, -57.042º	288
H. taragui	LJAMM-CNP 14419	ARG	Corrientes, Paraje Tres Cerros, Cerro Capará	-29.112º, -56.919º	147
	LJAMM-CNP 14420	ARG	Corrientes, Paraje Tres Cerros, Cerro Capará	-29.112º, -56.919º	147
H. underwoodi	LJAMM-CNP 10923	ARG	San Juan, Baños del Salado, 5 km N of Baños de la Laja	-31.313º, -68.446º	641
	LJAMM-CNP 10931	ARG	San Juan, 5.5 km E of Caucete	-31.662º, -68.209º	580
H. uruguayensis	UFRGS 1568	BRA	Rio Grande do Sul, Rosário do Sul	-30.2º, -54.9º	
	UFRGS 2139	BRA	Rio Grande do Sul, Rosário do Sul	-30.2º, -54.9º	
	UFRGS 2140	BRA	Rio Grande do Sul, Rosário do Sul	-30.2º, -54.9º	
	UFRGS 2579	BRA	Rio Grande do Sul, Rosário do Sul	-30.2º, -54.9º	
	UFRGS 2580	BRA	Rio Grande do Sul, Rosário do Sul	-30.2º, -54.9º	
	UFRGS 3929	BRA	Rio Grande do Sul, Rosário do Sul	-30.2º, -54.9º	
H. whitii	LJAMM-CNP 12110	ARG	Catamarca, W of Animan	-29.358º, -63.949º	410
	LJAMM-CNP 12111	ARG	Catamarca, W of Animan		

Species	Voucher	Country	Specific locality	Lat/Long	Elevation
	LJAMM-CNP 14387	ARG	Córdoba, Tulumba, RP 18, 9.5 km N of intersection with RP 16	-30.332º, -64.207º	907
	LJAMM-CNP 14388	ARG	Córdoba, Tulumba, RP 18, 9.5 km N of intersection with RP 16	-30.332º, -64.207º	907
H. williamsii	LJAMM-CNP 4467[3]	ARG	Buenos Aires, Tornquist, Parque Provincial Ernesto Tornquist	-38.055º, -62.014º	822
	LJAMM-CNP 6517	ARG	Buenos Aires, Tornquist, Parque Provincial Ernesto Tornquist	-38.136º, -61.986º	589
	LJAMM-CNP 6518	ARG	Buenos Aires, Tornquist, Parque Provincial Ernesto Tornquist	-38.136º, -61.986º	589
Garthia gaudichaudi	IBE-G1(1)	CHI	Unknown locality		
	IBE-G1(2)	CHI	Unknown locality		
Gymnodactylus geckoides	LG 911	BRA	Alagoas, Xingo		
	LG 1050	BRA	Sergipe, Barra dos Coqueiros	-10.9º, -37.0º	
Phyllodactylus kofordi	TG 266	PER	Chongoyape, Lambayeque	-6.7º, -79.9º	
Phyllopezus pollicaris	CHUNB 57388	BRA	Ceara, Tianguá	-3.7º, -40.9º	
Phyllopezus przewalskii	LJAMM-CNP 12089	ARG	Chaco, Fuerte Esperanza	-25.160º, -61.844º	169

Appendix S3

GenBank accession numbers (multi-genes)

GenBank accession numbers for each gene, and numbers of new sequences highlighted in bold. MD indicates missing data. Specimens with superindexes ([1–3]) are currently in BYU: 1- BYU 47960, 2- BYU 47941, 3- 47931.

Species	Voucher	ANL-19b	ANL-30b	RBMX	NKTR	SINCAIP	MXRA5	ACA4	DMXL1	PRLR	12S	Cytb
H. andicola	LJAMM-CNP 12490	KJ484255	KJ484235	KJ484346	KJ484394	KJ484369	KJ484348	KJ484324	KJ484298	KJ484274	MD	MD
	LJAMM-CNP 12493	MD	MD	MD	MD	MD	MD	MD	MD	MD	KJ484211	KJ484188
	LJAMM-CNP 12494	MD	MD	MD	MD	MD	MD	KJ484325	MD	MD	MD	MD
	LJAMM-CNP 12495	KJ484256	KJ484236	KJ484347	KJ484395	MD	KJ484349	MD	KJ484299	KJ484275	KJ484212	KJ484189
H. borellii	LJAMM-CNP 5842[1]	MD	MD	MD	MD	KJ484370	MD	MD	MD	MD	MD	MD
	LJAMM-CNP 12116	KJ484257	KJ484237	KJ484411	KJ484396	MD	KJ484350	KJ484326	KJ484300	KJ484276	KJ484213	KJ484205
	LJAMM-CNP 12125	KJ484258	KJ484238	KJ484412	KJ484397	KJ484393	KJ484351	KJ484327	KJ484301	KJ484277	KJ484214	KJ484206
H. darwinii	LJAMM-CNP 9266	KJ484260	KJ484240	KJ484414	KJ484409	KJ484372	KJ484352	KJ484329	KJ484303	MD	MD	KJ484191
	LJAMM-CNP 9813	MD	MD	MD	MD	MD	MD	MD	MD	KJ484278	MD	MD
	LJAMM-CNP 10638	MD	MD	MD	MD	MD	MD	MD	MD	KJ484295	MD	MD
	LJAMM-CNP 11424	MD	KJ484239	KJ484413	KJ484408	KJ484371	KJ484367	KJ484328	KJ484302	MD	KJ484215	KJ484190
	LJAMM-CNP 11432	KJ484259	MD	MD	MD	MD	MD	MD	MD	MD	KJ484216	MD
Homonota sp. A	LJAMM-CNP 5047[2]	KJ484262	KJ484242	KJ484416	MD	KJ484374	KJ484354	MD	KJ484305	KJ484280	KJ484218	KJ484192
	LJAMM-CNP 10505	MD	MD	MD	MD	KJ484373	MD	KJ484330	MD	MD	MD	MD
	LJAMM-CNP 10528	MD	MD	MD	KJ484399	MD	MD	KJ484331	MD	MD	MD	MD
	LJAMM-CNP 10577	KJ484261	KJ484241	KJ484415	KJ484398	MD	KJ484353	MD	KJ484304	MD	KJ484217	KJ484208
	LJAMM-CNP 12415	MD	MD	MD	MD	MD	MD	MD	MD	KJ484279	MD	MD

Species	Voucher	ANL-19b	ANL-30b	RBMX	NKTR	SINCAIP	MXRA5	ACA4	DMXL1	PRLR	12S	Cytb
Homonota sp. B	MNHNP 11406	**MF278837**	**MF278839**	**MF278851**	**MF278847**	**MF278853**	**MF278845**	**MF278841**	MD	**MF278849**	**MF278835**	**MF278843**
	MNHNP 11409	**MF278838**	**MF278840**	**MF278852**	**MF278848**	**MF278854**	**MF278846**	**MF278842**	**MF535517**	**MF278850**	**MF278836**	**MF278844**
H. rupicola	RUPI-1	KJ484263	KJ484243	KJ484426	MD	KJ484375	MD	MD	KJ484306	KJ484281	KJ484219	KJ484193
	RUPI-2	KJ484264	KJ484244	KJ484427	KJ484410	KJ484376	MD	MD	KJ484320	KJ484282	KJ484220	KJ484194
H. taragui	LJAMM-CNP 14419	KJ484265	KJ484245	KJ484428	MD	KJ484377	MD	KJ484332	KJ484321	KJ484283	KJ484221	KJ484195
	LJAMM-CNP 14420	KJ484266	KJ484246	KJ484429	KJ484400	KJ484378	MD	KJ484333	KJ484322	KJ484284	KJ484222	KJ484196
H. underwoodi	LJAMM-CNP 10923	KJ484269	KJ484249	MD	KJ484402	KJ484381	KJ484356	KJ484335	KJ484308	KJ484286	MD	KJ484197
	LJAMM-CNP 10931	KJ484270	KJ484250	MD	KJ484403	KJ484382	KJ484357	MD	KJ484309	KJ484297	MD	KJ484198
H. uruguayensis	UFRGS 1568	KJ484267	KJ484247	KJ484430	KJ484401	KJ484381	KJ484358	KJ484334	KJ484307	KJ484285	KJ484223	MD
	UFRGS 2139	MD	MD	MD	MD	KJ484382	MD	MD	MD	KJ484296	MD	MD
	UFRGS 2140	MD	MD	MD	MD	MD	MD	MD	MD	MD	KJ484224	MD
	UFRGS 2579	KJ484268	KJ484248	KJ484359	MD	MD	MD	MD	MD	MD	MD	MD
	UFRGS 2580	MD	MD	MD	MD	MD	MD	MD	MD	MD	MD	KJ484202
	UFRGS 3929	MD	MD	MD	MD	MD	MD	MD	KJ484323	MD	MD	MD
H. whitii	LJAMM-CNP 12110	MD	KJ484251	KJ484360	MD	KJ484383	KJ484359	KJ484336	MD	MD	MD	MD
	LJAMM-CNP 12111	KJ484271	MD	MD	MD	MD	KJ484360	KJ484337	MD	MD	MD	MD
	LJAMM-CNP 14387	MD	MD	MD	KJ484404	MD	MD	MD	KJ484310	MD	KJ484225	KJ484203
	LJAMM-CNP 14388	MD	KJ484252	KJ484432	KJ484405	KJ484384	MD	MD	KJ484311	MD	KJ484226	KJ484204
H. williamsii	LJAMM-CNP 4467[3]	KJ484272	KJ484253	KJ484417	MD	KJ484385	KJ484360	KJ484338	KJ484312	KJ484287	KJ484227	KJ484205
	LJAMM-CNP 6517	KJ484273	KJ484254	KJ484418	KJ484407	KJ484386	KJ484361	KJ484339	KJ484313	KJ484288	KJ484228	KJ484206
	LJAMM-CNP 6518	MD	MD	MD	KJ484406	MD	MD	MD	MD	MD	MD	MD
Garthia gaudichaudi	IBE-G1(1)	MD	MD	KJ484419	MD	KJ484387	KJ484362	MD	KJ484314	KJ484289	KJ484229	MD
	IBE-G1(2)	MD	MD	KJ484420	MD	MD	MD	KJ484340	MD	MD	KJ484230	MD
Gymnodactylus geckoides	LG 911	MD	MD	KJ484421	MD	KJ484389	KJ484364	KJ484342	KJ484316	KJ484291	KJ484232	KJ484210
	LG 1050	MD	MD	KJ484431	MD	KJ484388	KJ484363	KJ484341	KJ484315	KJ484290	KJ484231	KJ484209

Species	Voucher	ANL-19b	ANL-30b	RBMX	NKTR	SINCAIP	MXRA5	ACA4	DMXL1	PRLR	12S	Cytb
Phyllodactylus kofordi	TG 266	MD	MD	KJ484422	MD	KJ484390	KJ484368	KJ484343	KJ484317	KJ484292	KJ484233	KJ484207
Phyllopezus pollicaris	CHUNB 57388	MD	MD	KJ484424	MD	KJ484392	KJ484366	KJ484345	KJ484319	KJ484294	KJ484234	KJ484204
Phyllopezus przewalskii	LJAMM-CNP 12089	MD	MD	KJ484423	MD	KJ484391	KJ484365	MD	KJ484318	KJ484293	MD	KJ484203

Appendix S4

Primers used for genetic analyses

Primers' sequences for each gene, used for analyses. For every primer we indicate the author, and the full reference is provided below the table. We maintain the original name of the primers, providing an indication for forward (F) and reverse (R) primers in square brackets when not specified in the original name.

Gene	Primer	Sequence (5' -> 3')	Author
16S	L2510 [F]	GCCTGTTTAACAAAAACAT	Miya & Nishida 1996
	H3056 [R]	CGGTCTG AACTCAGATCACGT	Miya & Nishida 1996
12S	tPhe [F]	AGCACRGCACTGA	Wiens et al. 1999
	12e [R]	RCGCTTACCWTGTTAC	Wiens et al. 1999
Cytb	CB3	GGCAAATAGGAARTATCATTC	Palumbi 1996
	CB3R	CATATTAAACCCGAATGATAYTT	Palumbi 1996
ANL-19b	Homo19b_F	CCTAAGAAAAGAGAAGGCAATTCA	Morando et al. 2014
	Homo19b_R	TGCATGCTACTCAGATTCCTG	Morando et al. 2014
ANL-30b	Homo30b_F	CAATCCAGTCGAAGGAAGGA	Morando et al. 2014
	Homo30b_R	AAACTTGGTTGGGTGCAGAG	Morando et al. 2014
RBMX	HNRNP1F	CCACGAGATTATGCCTACCG	Gamble et al. 2011
	HNRNP1R	CATCATAKCGACTGCTTCCA	Gamble et al. 2011
	RBMX-F1	TCCTCTTACAGTGAYCGTGATG	Gamble et al. 2011

	RBMX-R1	TCCCGTAATCATCATAGCGACT	Gamble et al. 2011
NKTR	NTKRf1	AGTAAATGGGAYTCKGARTCAAA	Townsend et al. 2011
	NKTRr3	KCGTGCYGTCTTYCTWACTTCA	Townsend et al. 2011
SINCAIP	SNCAIP_f10	CGCCAGYTGYTGGGRAARGAWAT	Townsend et al. 2008
	SNCAIP_r13	GGWGAYTTGAGDGCACTCTTRGGRCT	Townsend et al. 2008
MXRA5	MXRA5_F1	YATTTTGGCAAARGTCCGTGGGAARA	Portik et al. 2012.
	MXRA5_F2	KGCTGAGCCTKCCTGGGTGA	Portik et al. 2012.
	MXRA5_R1	WTGTGCTGCATATGCTGTWATCTCWGGT	Portik et al. 2012.
	MXRA5_R2	YCTMCGGCCYTCTGCAACATTK	Portik et al. 2012.
ACA4	ACA4 [F]	GAGCGTGGCTAYTCCTTTGT	Waltari & Edwards 2002
	ACA4 [R]	GTGGCCATTTCATTCTCAAA	Waltari & Edwards 2002
DMXL1	DMXL1(F2)	GTCTAGGGAGGATGGTTCACATA	Werneck et al. 2012
	DMXL1(R2)	GAATGAAGCAAGTGACSAGAAAGA	Werneck et al. 2012
PRLR	PRLR_f1	GACARYGARGACCAGCAACTRATGCC	Townsend et al. 2008
	PRLR_r3	GACYTTGTGRACTTCYACRTAATCCAT	Townsend et al. 2008

References

Gamble T, Daza JD, Colli RC, Vitt LJ, Bauer AM. 2011. A new genus of miniaturized and pug-nosed gecko from South America (Sphaerodactylidae: Gekkota). Zoological Journal of the Linnean Society, 163(4): 1244–1266. doi: 10.1111/j.1096-3642.2011.00741.x

Miya M, Nishida M. 1996. Molecular phylogenetic perspective on the evolution of the deep-sea fish genus *Cyclothone* (Stomiiformes: Gonostomatidae). *Ichthyological Research* 43(4):375–398. doi: 10.1007/BF02347637

Morando M, Medina CD, Ávila LJ, Pérez CHF, Buxton A, Sites JW. 2014. Molecular phylogeny of the New World gecko genus *Homonota* (Squamata: Phyllodactylidae). *Zoologica Scripta* 43(3):249–260. doi: 10.1111/zsc.12052

Palumbi SR. 1996. Nucleic Acids II: The Polymerase Chain Reaction. In: Hillis DM, Moritz C, Mable BK, eds. *Molecular Systematics, 2nd ed.* Massachusetts: Sinauer Associates, Inc., 205–247.

Portik DM, Wood PL, Grismer JL, Stanley EL, Jackman TR. 2012. Identification of 104 rapidly-evolving nuclear protein-coding markers for amplification across scaled reptiles using genomic resources. *Conservation Genetics Resources* 4(1):1–10. doi: 10.1007/s12686-011-9460-1

Townsend TM, Alegre RE, Kelley ST, Wiens JJ, Reeder TW. 2008. Rapid development of multiple nuclear loci for phylogenetic analysis using genomic resources: An example from squamate reptiles. *Molecular Phylogenetics and Evolution* 47(1):129–142. http://doi.org/10.1016/j.ympev.2008.01.008

Townsend TM, Mulcahy DG, Noonan BP, Sites JW, Kuczynski CA, Reeder TW. 2011. Phylogeny of iguanian lizards inferred from 29 nuclear loci, and a comparison of concatenated and species-tree approaches for an ancient, rapid radiation. *Molecular Phylogenetics and Evolution* 61(2):363–380. doi: 10.1016/j.ympev.2011.07.008

Waltari E, Edwards SV. 2002. Evolutionary dynamics of intron size, genome size, and physiological correlates in archosaurs. *American Naturalist* 160(5):539–552. doi: 10.1086/342079

Werneck FP, Gamble T, Colli GR, Rodrigues MT, Sites JW. 2012. Deep diversification and long term persistence in the South American “Dry Diagonal”. Integrating continent-wide phylogeography and distribution modeling of geckos. *Evolution* 66(10):3014–3034. doi: 10.1111/j.1558-5646.2012.01682.x

Wiens JJ, Reeder TW, Nieto Montes de Oca A. 1999. Molecular phylogenetics and evolution of sexual dichromatism among populations of the Yarrow’s spiny lizard (*Sceloporus jarrovii*). *Evolution* 53(6):1884–1897. doi: 10.2307/2640448

Appendix S5

Examined specimens for morphology

Specimens used for morphological comparison. Specimens marked with "[H]" and "[P]" indicate holotype and paralectotypes of *Gymnodactylus horridus* respectively. Codes for countries are: ARG (Argentina), BOL (Bolivia), and PAR (Paraguay). Locality information is provided from the major political division of the country to the most specific location data. References to routes are RP (Provincial Route) and RN (National Route). Coordinates given with only one decimal are approximately, in which case elevation is not provided.

Specimen	Country	Specific locality	Lat/Long	Elevation
IZH-R 1 [H]	ARG	Mendoza, Canyons of the Sierra del Challao	-32.9º, -68.9º	
IZH-R 2 [P]	ARG	Mendoza, Canyons of the Sierra del Challao	-32.9º, -68.9º	
IZH-R 3 [P]	ARG	Mendoza, Canyons of the Sierra del Challao	-32.9º, -68.9º	
LJAMM-CNP 6520	ARG	Río Negro, Villa Regina, fosa del tiro federal	-39.096º, -67.051º	214
LJAMM-CNP 6530	ARG	Río Negro, Villa Regina, bardas	-39.092º, -67.076º	261
LJAMM-CNP 6532	ARG	Río Negro, Villa Regina, bardas	-39.092º, -67.076º	261
LJAMM-CNP 6533	ARG	Río Negro, Villa Regina, bardas	-39.092º, -67.076º	261
LJAMM-CNP 6535	ARG	Río Negro, Villa Regina, bardas	-39.092º, -67.076º	261
LJAMM-CNP 6967	ARG	Neuquén, Villa El Chocón, N margin of Limay river	-39.254º, -68.781º	469
LJAMM-CNP 6968	ARG	Neuquén, Villa El Chocón	-39.254º, -68.781º	469

Specimen	Country	Specific locality	Lat/Long	Elevation
LJAMM-CNP 7670	ARG	Río Negro, Avellaneda, Chimpay	-39.145º, -66.145	157
LJAMM-CNP 7674	ARG	Río Negro, Avellaneda, Chimpay	-39.145º, -66.145	157
LJAMM-CNP 7804	ARG	Neuquén, RP 5, 10 km N from RP 7	-37.906º, -69.174º	555
LJAMM-CNP 8713	ARG	Neuquén, RP 7, 41 km NW Punta Carranza	-37.656º, -69.472º	662
LJAMM-CNP 10493	ARG	Mendoza, RP 190, 1 km S Punta de Agua	-35.541º, -68.079º	789
LJAMM-CNP 10496	ARG	Mendoza, RP 190, 1 km S Punta de Agua	-35.541º, -68.079º	789
LJAMM-CNP 10523	ARG	La Pampa, RP 1, 23.6 km W intersection with RN 151	-37.075º, -67.785º	542
LJAMM-CNP 10526	ARG	La Pampa, RP 1, 23.6 km W intersection with RN 151	-37.075º, -67.785º	542
LJAMM-CNP 10576	ARG	Mendoza, RP 190, 1 km S Punta de Agua	-35.541º, -68.079º	789
LJAMM-CNP 10577	ARG	Mendoza, RP 190, 1 km S Punta de Agua	-35.541º, -68.079º	789
LJAMM-CNP 10578	ARG	La Pampa, RP 27, 37.7 km S intersection with RP 14	-36.668º, -68.022º	766
LJAMM-CNP 10579	ARG	La Pampa, RP 27, 37.7 km S junction with RP 14	-36.668º, -68.022º	766
LJAMM-CNP 10584	ARG	La Pampa, RP 1, 23.6 km W intersection with RN 151	-37.075º, -67.785º	542
LJAMM-CNP 13948	ARG	Neuquén, RN 237, 6 km SW Picun Leufu	-39.555º, -69.301º	431
LJAMM-CNP 14551	ARG	Neuquén, Mina La Casualidad	-37.904º, -68.488º	462
MNHNP 2821	PAR	Boquerón, Parque Nacional Teniente Enciso	-21.209º, -61.655	253

Specimen	Country	Specific locality	Lat/Long	Elevation
MNHNP 9037	PAR	Boquerón, Parque Nacional Teniente Enciso	-21.209º, -61.655	253
MNHNP 9038	PAR	Boquerón, Parque Nacional Teniente Enciso	-21.209º, -61.655	253
MNHNP 9131	PAR	Boquerón, Parque Nacional Teniente Enciso	-21.209º, -61.655	253
MNHNP 11406	PAR	Boquerón, Parque Nacional Teniente Enciso	-21.209º, -61.655	253
MNHNP 11410	PAR	Boquerón, Parque Nacional Teniente Enciso	-21.209º, -61.655	253
MNHNP 11419	PAR	Boquerón, Parque Nacional Teniente Enciso	-21.209º, -61.655	253
MNHNP 11421	PAR	Boquerón, Parque Nacional Teniente Enciso	-21.209º, -61.655	253
MNHNP 11423	PAR	Boquerón, Parque Nacional Teniente Enciso	-21.209º, -61.655	253
MNHNP 11850	PAR	Boquerón, Cruce San Miguel, in front of Parque Nacional Teniente Enciso	-21.203º, -61.662º	254
MNHNP 11855	PAR	Boquerón, Cruce San Miguel, in front of Parque Nacional Teniente Enciso	-21.203º, -61.662º	254
MNHNP 11860	PAR	Boquerón, Cruce San Miguel, in front of Parque Nacional Teniente Enciso	-21.203º, -61.662º	254
MNHNP 11872	PAR	Boquerón, Cruce San Miguel, in front of Parque Nacional Teniente Enciso	-21.203º, -61.662º	254
MNHNP 12238	PAR	Boquerón, Fortín Mayor Infante Rivarola	-21.679º, -62.401º	277
SMF 29277	BOL	Tarija, Villamontes	-21.266º, -63.451º	398
SMF 101984	PAR	Boquerón, Fortín Mayor Infante Rivarola	-21.679º, -62.401º	277

Appendix S6

Results of ABGD analysis

Number of groups according to the prior intraspecific divergence. Color of "Initial Partitions" were modified from original for a better contrast. Under the graphic, is presented the list of partitions with suggested grouping and each maximal intraspecific divergence.

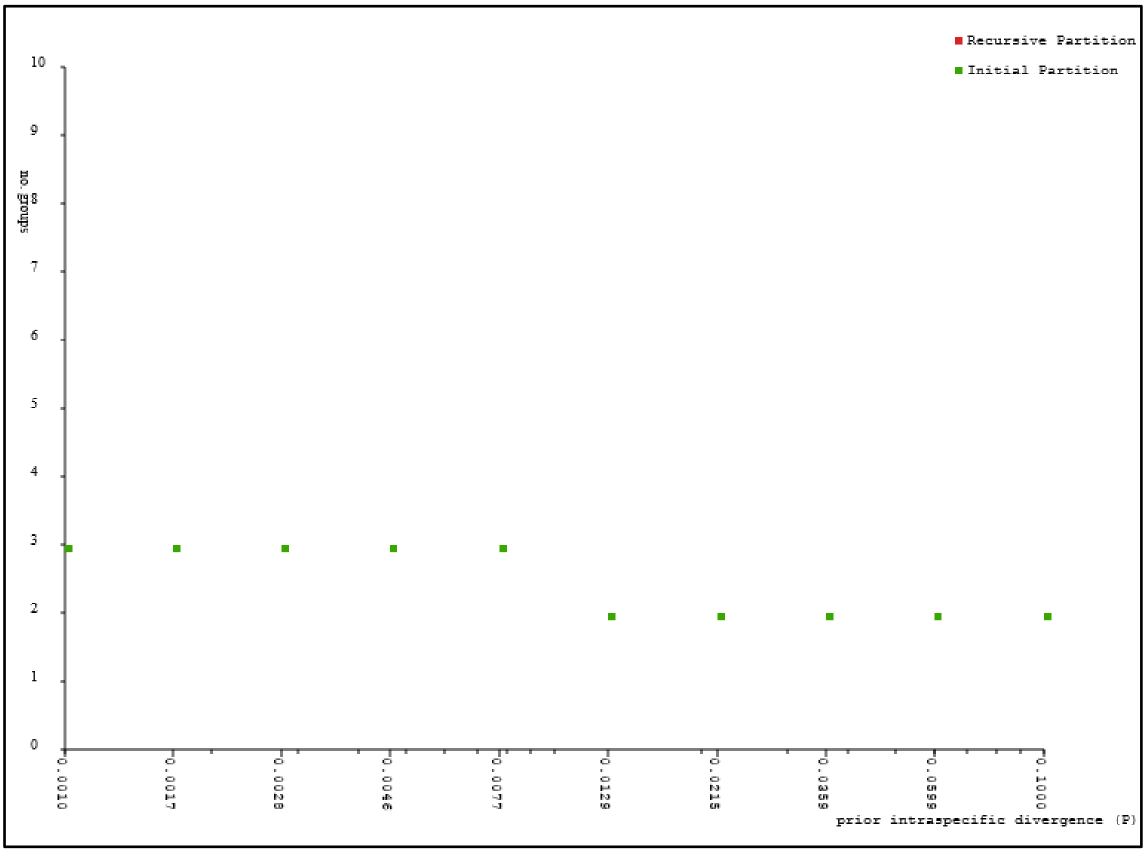

Partition 1 : found 3 groups (prior maximal distance P= 0.001000)

Partition 2 : found 3 groups (prior maximal distance P= 0.001668)

Partition 3 : found 3 groups (prior maximal distance P= 0.002783)

Partition 4 : found 3 groups (prior maximal distance P= 0.004642)

Partition 5 : found 3 groups (prior maximal distance P= 0.007743)

Partition 6 : found 2 groups (prior maximal distance P= 0.012915)

Partition 7 : found 2 groups (prior maximal distance P= 0.021544)

Partition 8 : found 2 groups (prior maximal distance P= 0.035938)

Partition 9 : found 2 groups (prior maximal distance P= 0.059948)

Partition 10 : found 2 groups (prior maximal distance P= 0.100000)

Groups detected when Initial Partition with prior maximal distance $P=7.74e^{-03}$

Group[1] n: 3 ;id: LJAMM-CNP_10495 LJAMM-CNP_10576 LJAMM-CNP_5047

Group[2] n: 3 ;id: MNHNP_11873 MNHNP_12238 SMF_101984

Group[3] n: 1 ;id: Phyllopezus_SMF-100495

Histogram of distances

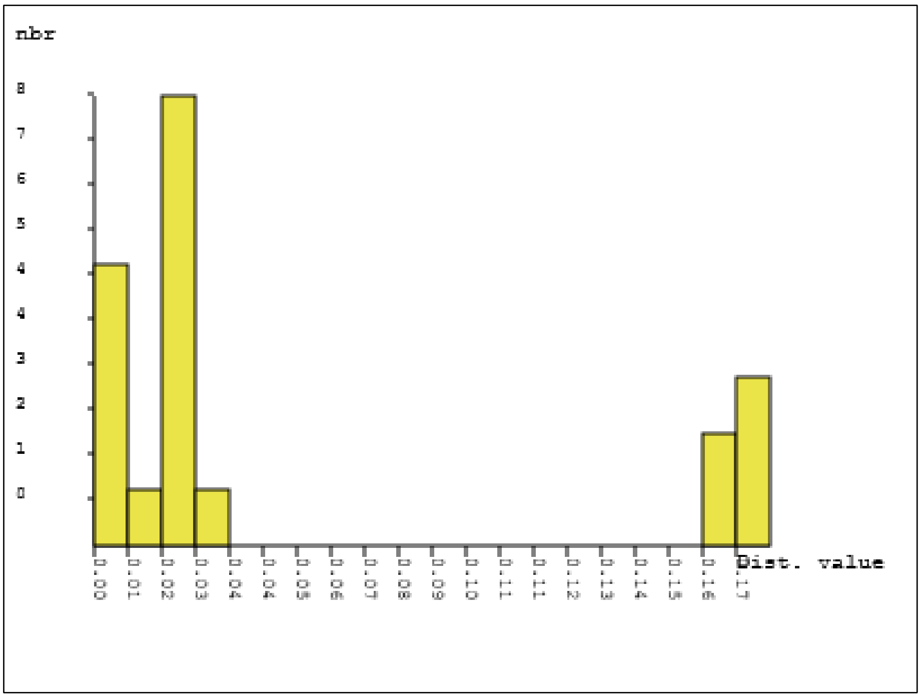

Ranked distances

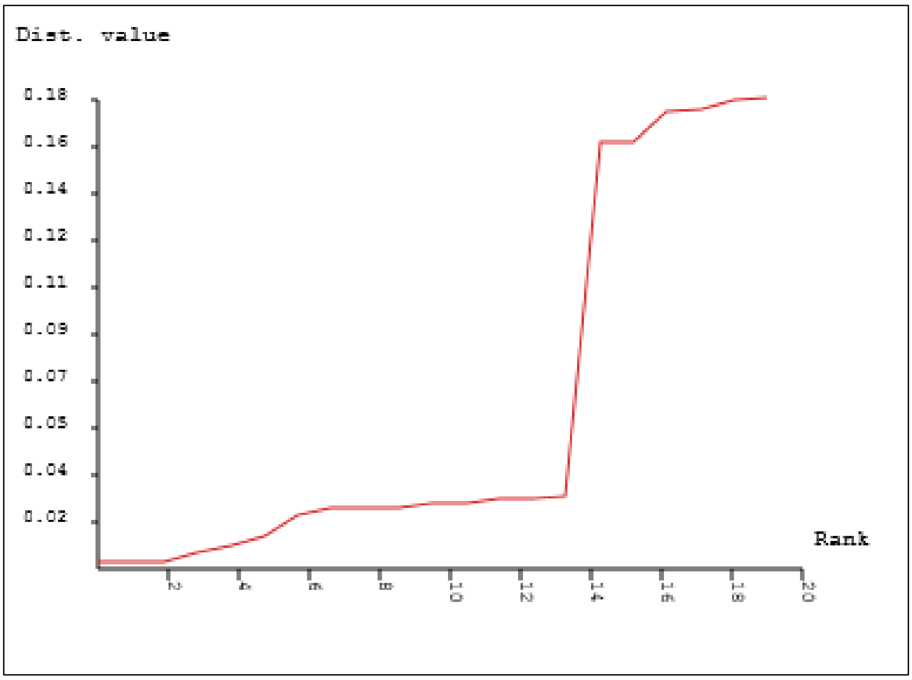

Appendix S7

Results of PTP analysis

ML solution produced with PTP. Numbers on nodes indicate support values. The red clades represent putative species. In this case, one species is composed of Argentinean samples (LJAMM-CNP 5047, 10495, 10576), and the other of Paraguayan specimens (MNHNP 11873, 12238, SMF 101984).

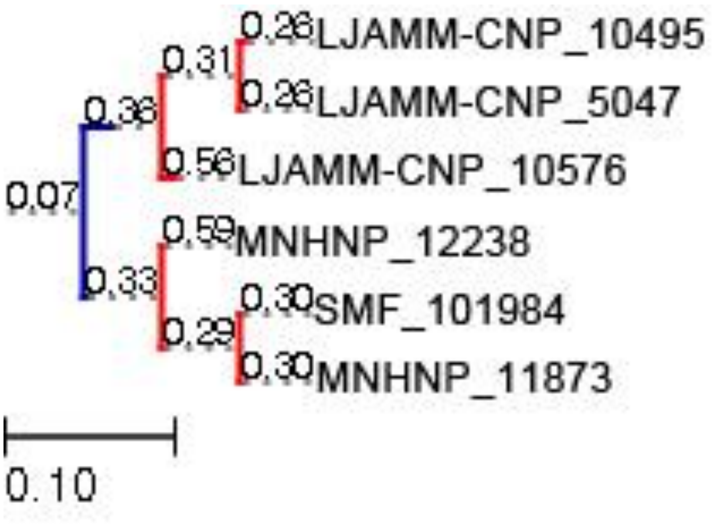

Appendix S8 - Individual gene trees (Explanations at the end)

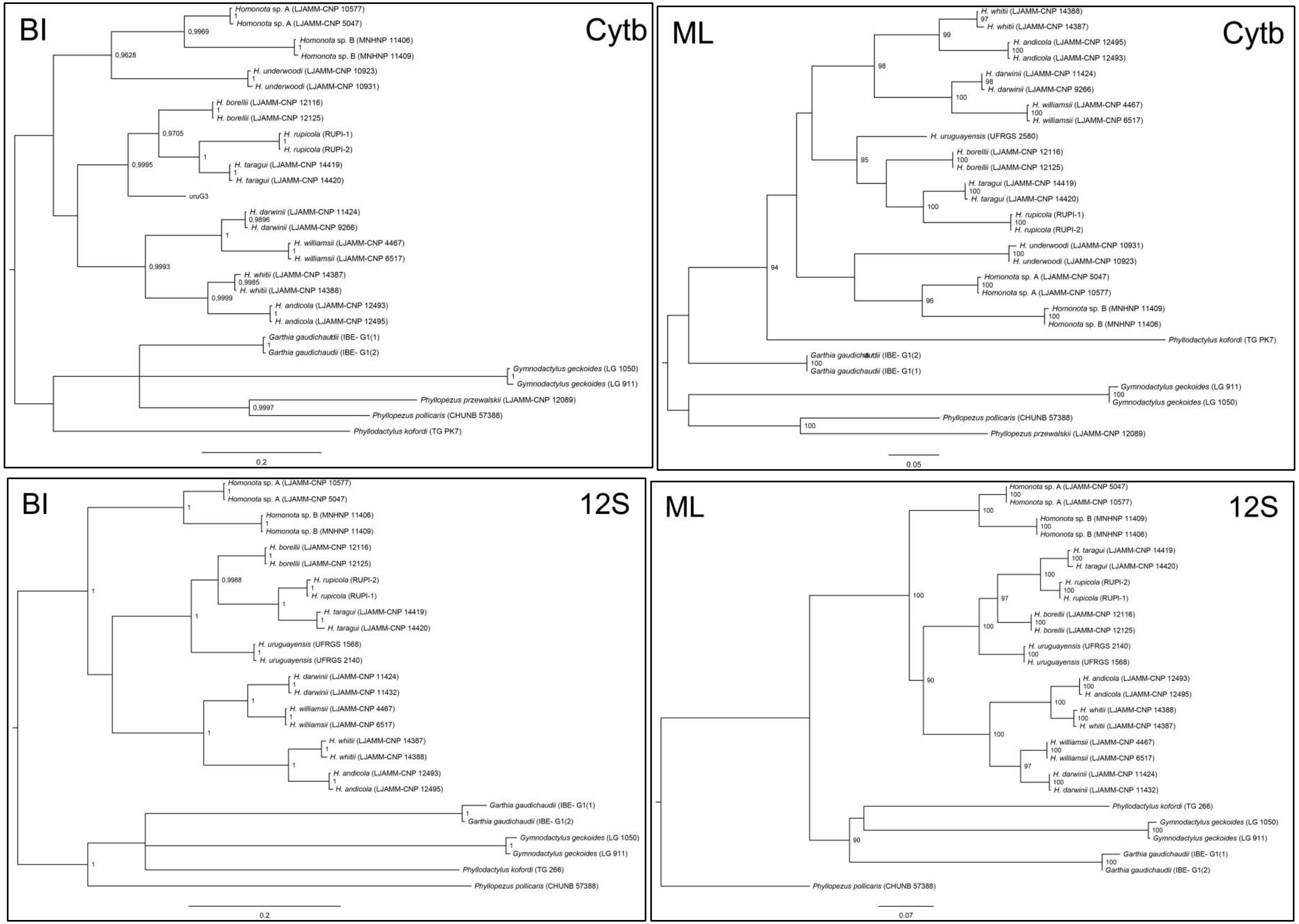

Appendix S8 (continuation)

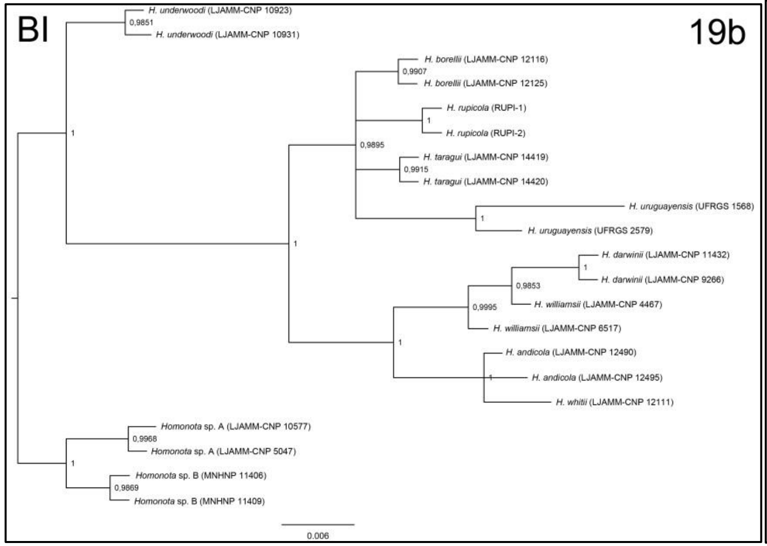

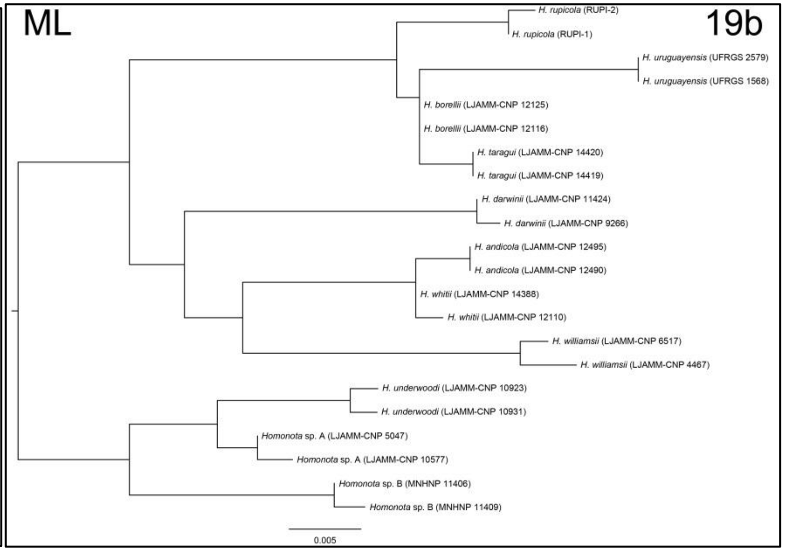

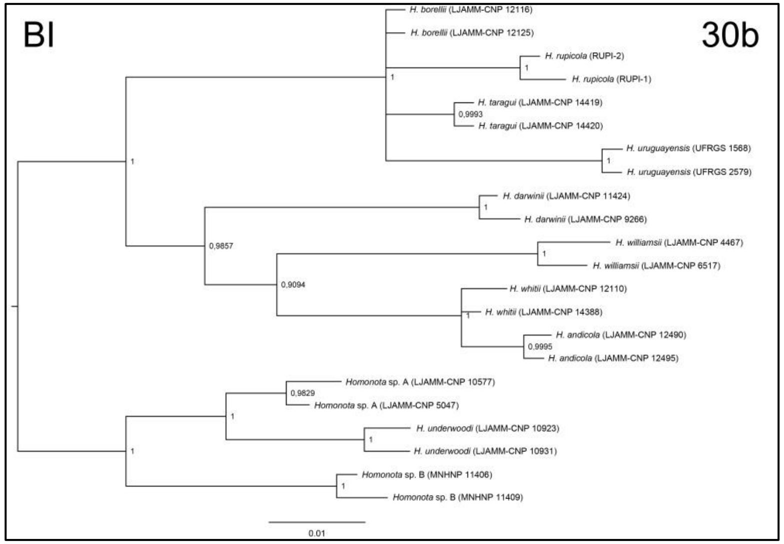

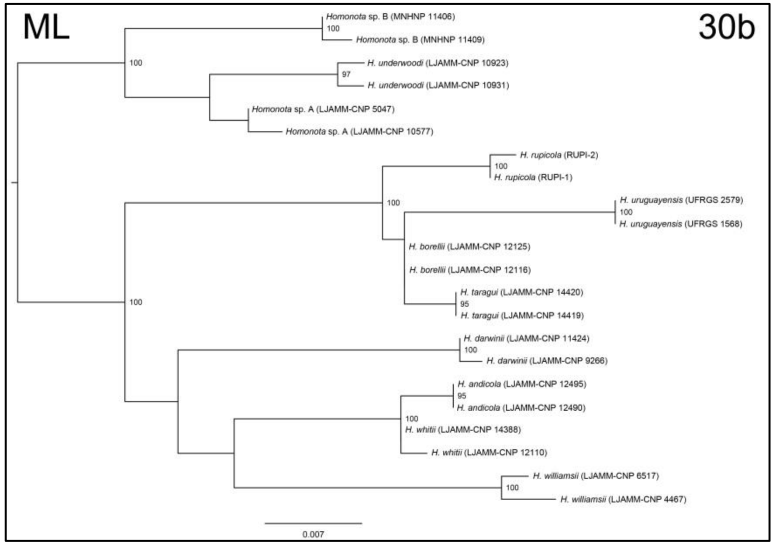

Appendix S8 (continuation)

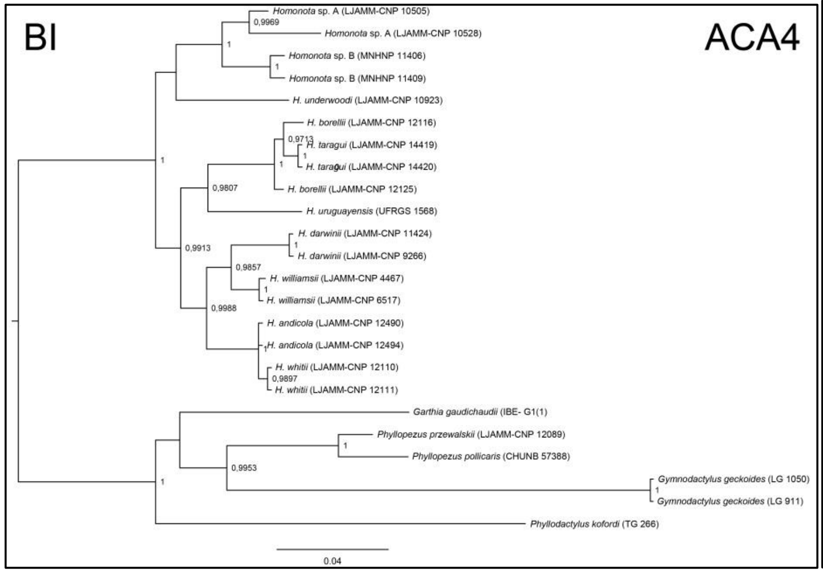

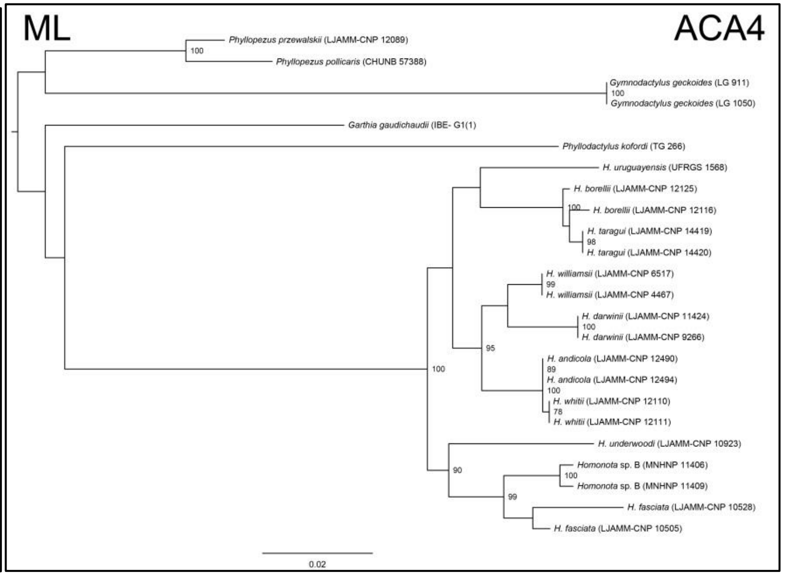

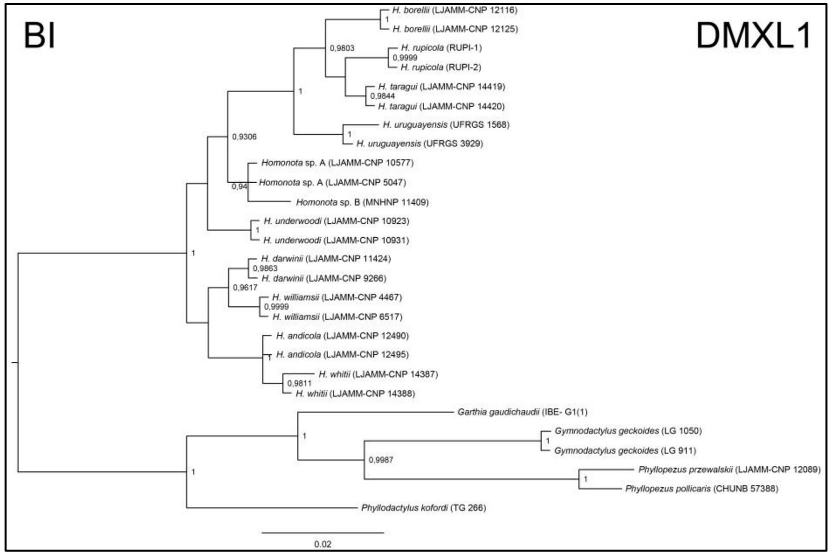

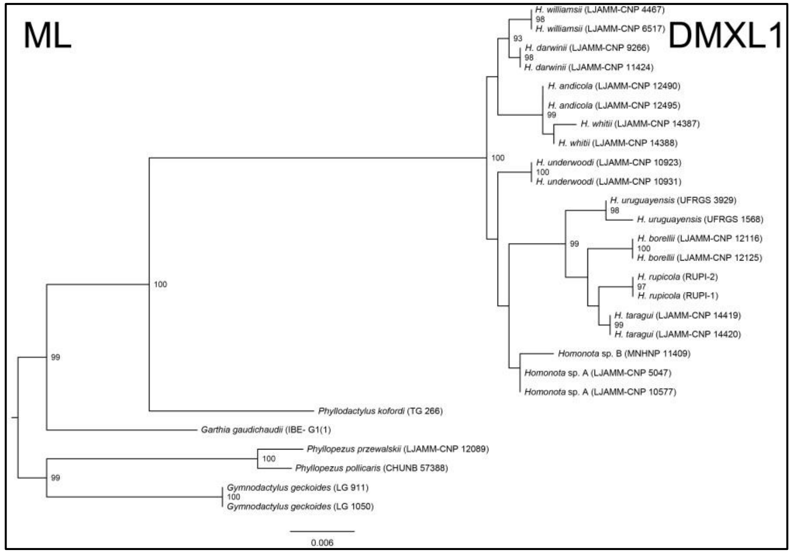

Appendix S8 (continuation)

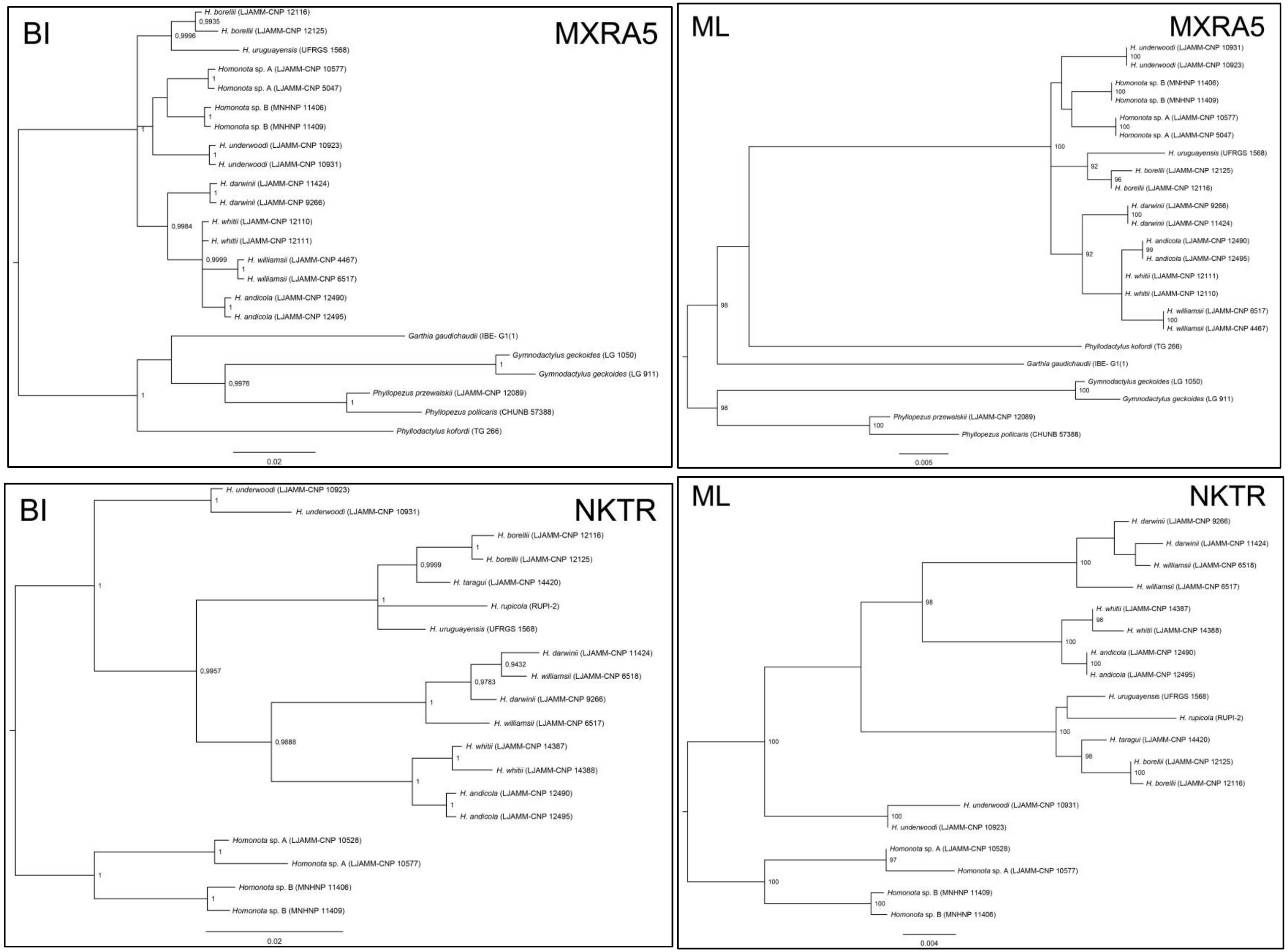

Appendix S8 (continuation)

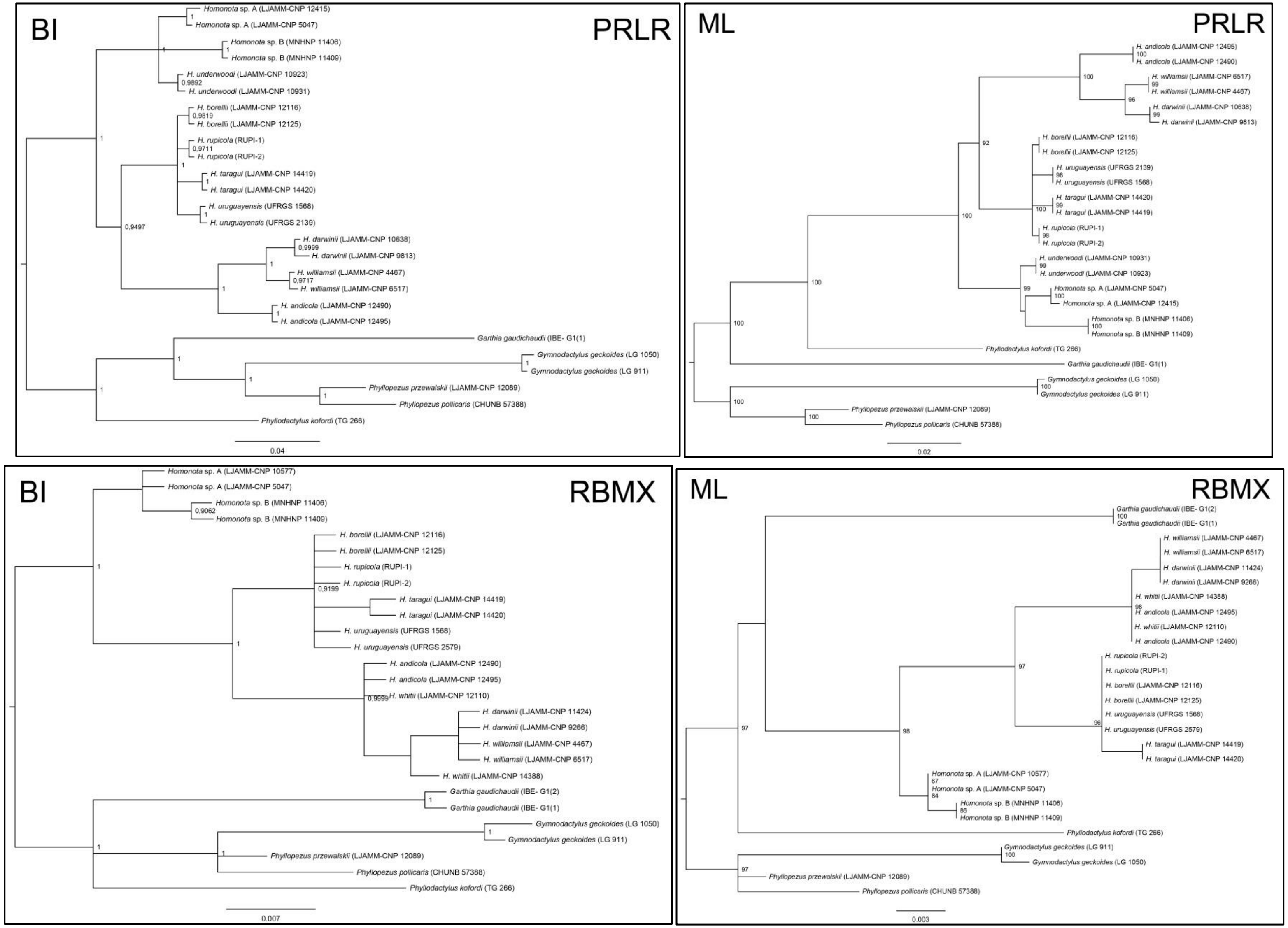

Appendix S8 (continuation)

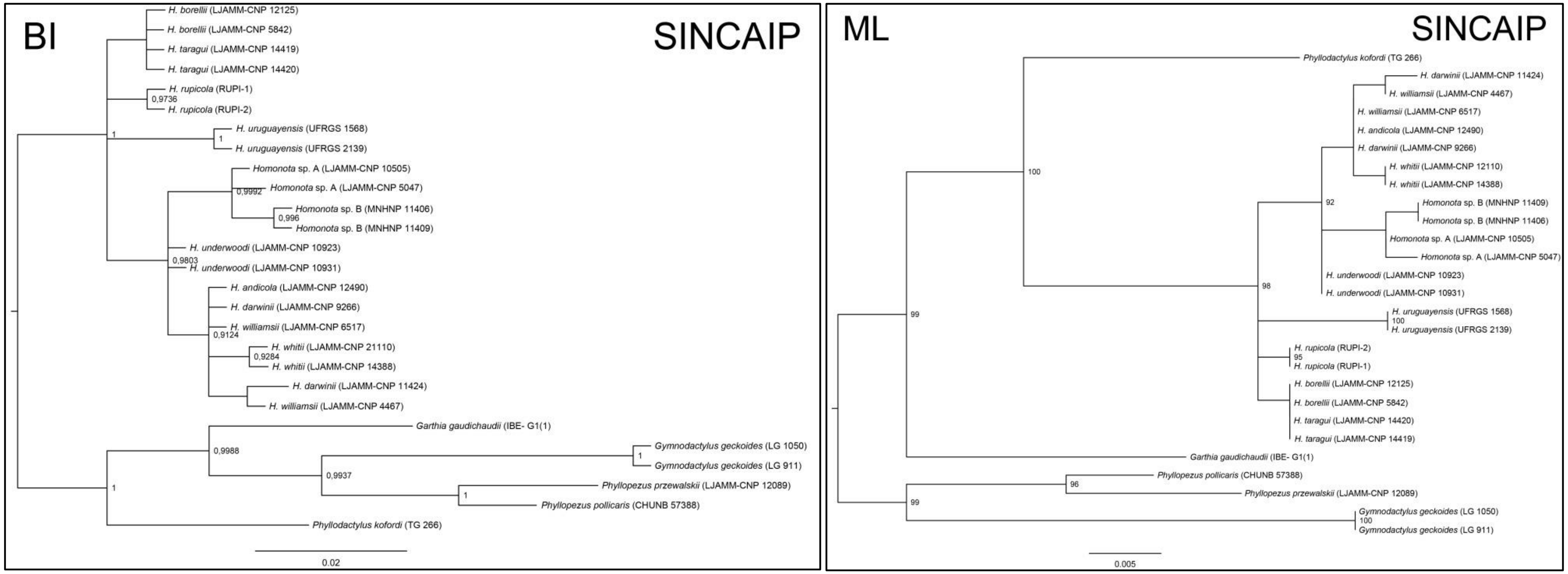

Individual gene trees generated by Bayesian analysis (BI) and Maximum Likelihood (ML). Acronyms for genes are cytb (cytochrome *b*), 12S (small ribosomal subunit), ACA4 (alpha-cardiac actin intron 4), DMXL1 (dmX-like protein 1), MXRA5 (encoding matrix remodeling associated intron 5), NKTR (natural killer-tumor recognition sequence), PRLR (prolactin receptor), RBMX (intron 8 and flanking exon regions of RNA binding motif protein), SINCAIP (synuclein alpha interacting protein), 19b and 30b (anonymous nuclear loci). The two first genes are mitochondrial, and the remaining nine are nuclear. Only values of ≥0.90 (for BI) and ≥90 (for ML) are shown.

Appendix S9 – Concatenated trees

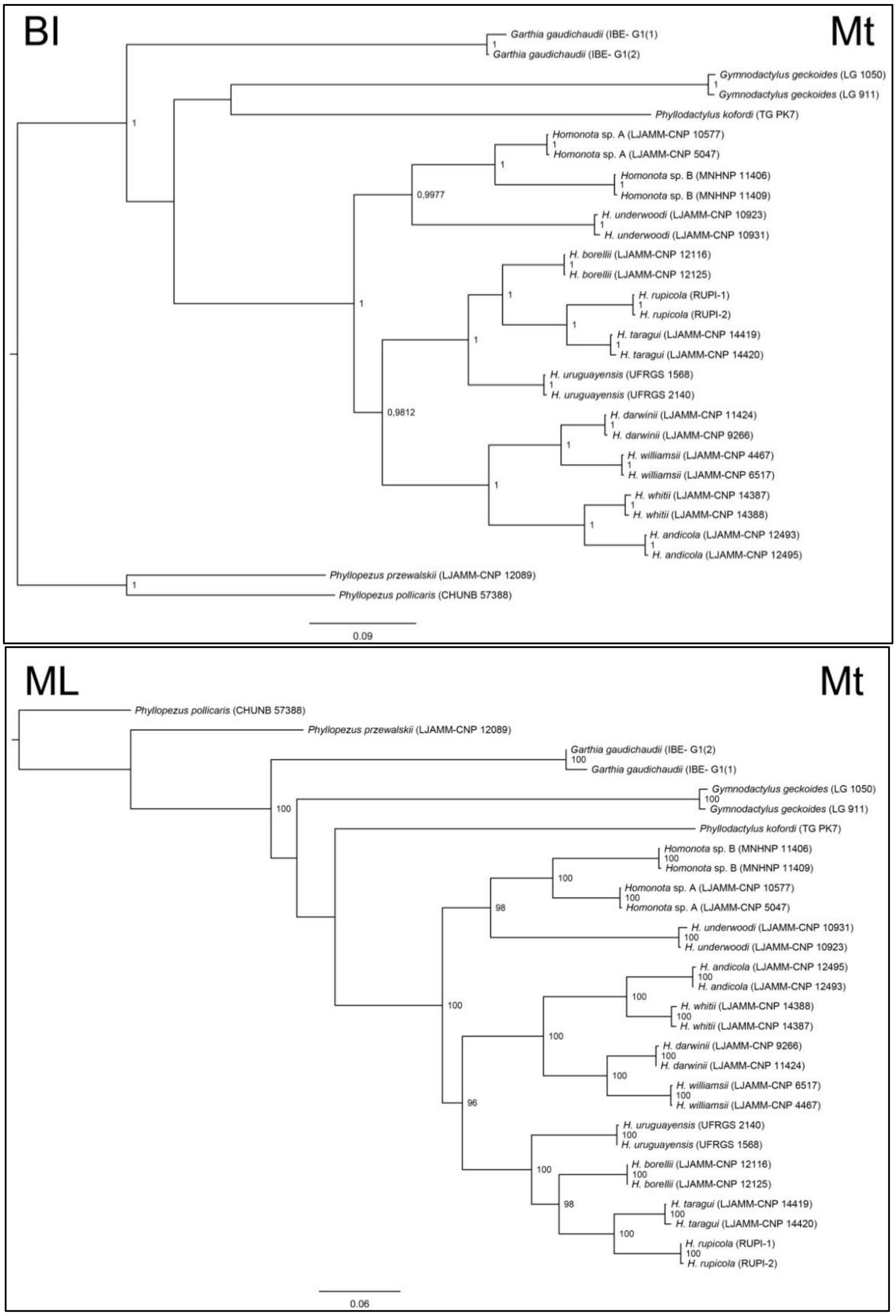

Bayesian (above) and Maximum Likelihood (below) trees inferred from concatenated mitochondrial genes. See Materials and Methods for indication of mitochondrial genes. Only values of ≥0.90 (for BI) and ≥90 (for ML) are shown.

Appendix S9 (continuation)

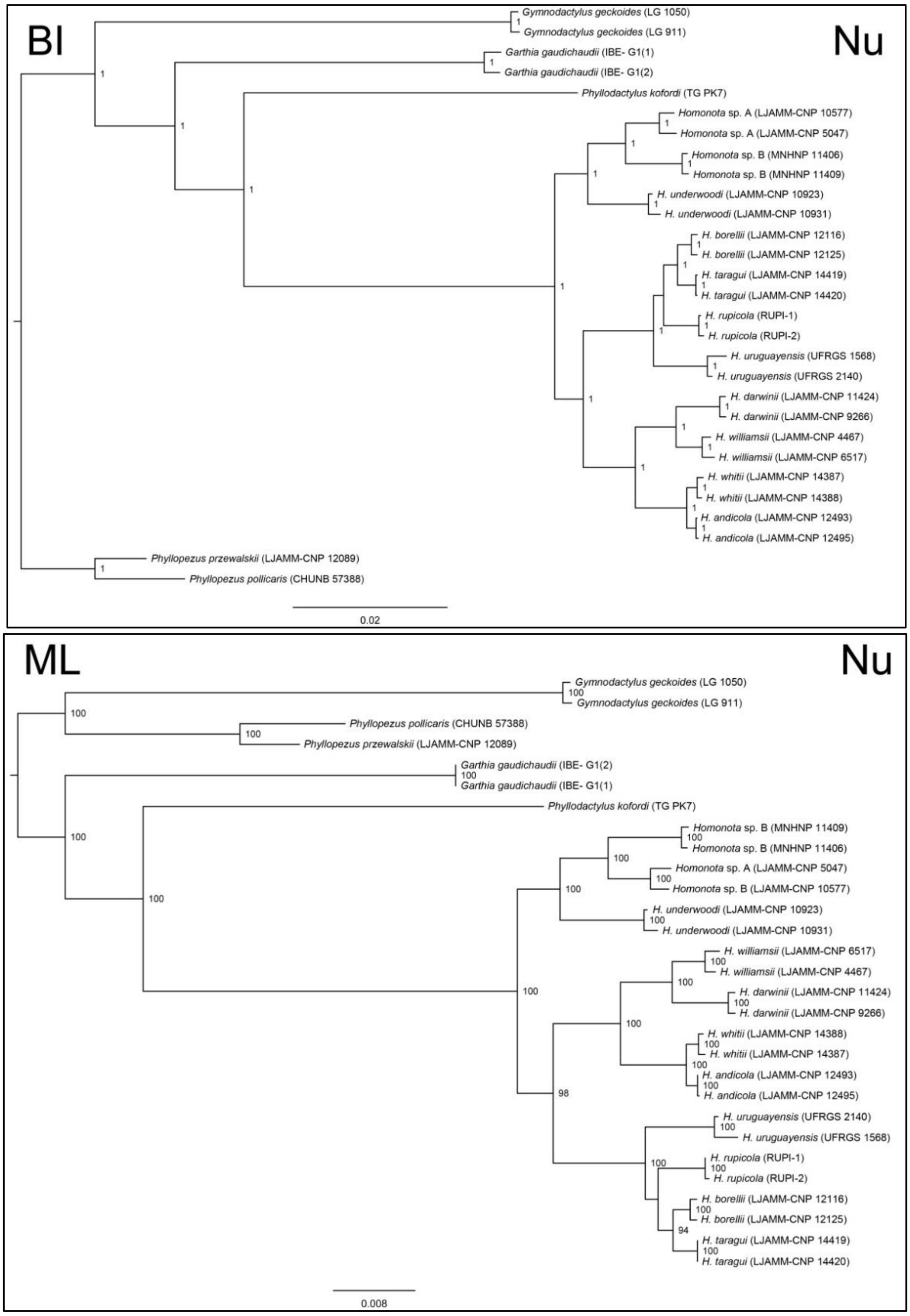

Bayesian (above) and Maximum Likelihood (below) trees inferred from concatenated nuclear genes. See Materials and Methods for indication of nuclear genes. Only values of ≥0.90 (for BI) and ≥90 (for ML) are shown.

Appendix S9 (continuation)

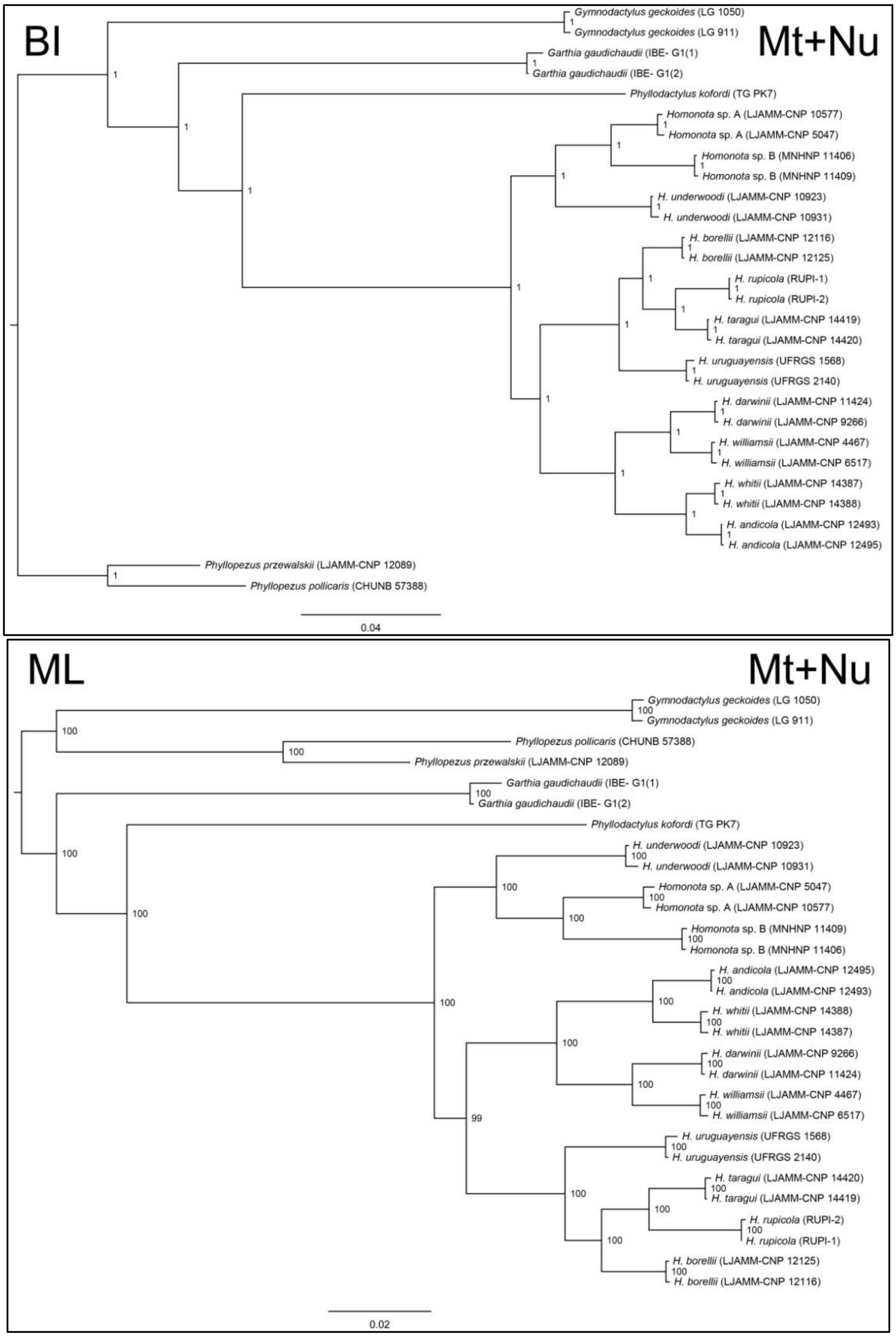

Bayesian (above) and Maximum Likelihood (below) trees inferred from concatenated mitochondrial and nuclear genes. Only values of ≥0.90 (for BI) and ≥90 (for ML) are shown.

Appendix S10

Most contributing variables for DA

A- Continuous variables

Contribution values for the three principal axes of the discriminant function analysis of continuous variables. Most highly contributing variables in bold. Abbreviations: AL (arm length), EMD (eye–meatus distance), END (eye–nostril distance), ESD (eye–snout distance), FL (foot length), HH (head height), HL (head length), HW (head width), ID (interorbital distance), IND (internostril distance), SVL (snout–vent length), TL (tibial length), TrL (trunk length). See "Morphological Approach" in the Materials and Methods section for details about measurements.

Variables	Axis 1	Axis 2	Axis 3
SVL	**-0.25403**	**2.77550**	**0.40997**
TrL	**-0.45427**	**1.26620**	0.25683
FL	-0.09004	0.50099	-0.07282
TL	0.06197	0.43801	0.05459
AL	0.03385	0.50497	**0.39286**
HL	-0.21662	0.47604	**0.37498**
HW	-0.21465	0.32961	0.33726
HH	-0.23825	0.11503	0.23913
END	-0.08492	0.14669	0.02613
ESD	-0.11675	0.25690	0.20150
EMD	0.02475	0.24455	0.21194
ID	-0.01922	0.17126	0.20578
IND	-0.06752	0.06858	0.05570

B- Discrete variables

Contribution values for the three principal axes of the discriminant function analysis of discrete variables. Most highly contributing variables in bold. Abbreviations: 3FL (third finger lamellae), 4TL (fourth toe lamellae), DT (dorsal tubercles), LVS (longitudinal rows of ventral scales), TVS (transversal rows of ventral scales). See "Morphological Approach" in the Materials and Methods section for details about counts.

Variables	Axis 1	Axis 2	Axis 3
DT	**1.08450**	0.23782	1.22050
TVS	0.67569	**-1.77600**	-0.65160
LVS	-0.34039	-0.87093	**1.46070**
4TL	0.59106	0.39061	0.33669
3FL	0.84406	-0.14672	0.42342

Appendix S11

Matrix of metric data

Raw metric data (in mm) used for analyses. See Materials and Methods section for explanation of the characters. Missing data indicated with "?". Argentinean populations belong to LJAMM-CNP collection, whereas Paraguayan specimens are housed in the MNHNP and SMF.

Specimen	SVL	TrL	FL	TL	AL	HL	HW	HH	END	ESD	EMD	ID	IND	Sex
LJAMM-CNP 6520	57	24	10	9.2	12.2	12.8	8.6	6.7	4.3	5.3	4.8	3.8	1.7	F
LJAMM-CNP 6530	63	29	11	12.5	15.4	14.6	10.4	7.6	4.7	6.3	5.3	4.8	1.7	F
LJAMM-CNP 6532	55	23	9	9.6	?	13.5	10.5	6.9	4.6	5.8	5.2	5.2	1.8	M
LJAMM-CNP 6533	59	25	?	11.2	13.4	13.9	11.1	7.1	4.8	6.1	4.9	4.9	2.3	F
LJAMM-CNP 6535	52	20	11	9.6	?	?	?	?	?	?	?	?	?	M
LJAMM-CNP 6967	41	18	7	8.3	11.9	10.5	10.4	5.8	3.5	4.1	4.4	4.5	1.7	M
LJAMM-CNP 6968	45	18	9	9.0	13.7	?	8.9	?	?	?	?	4.6	1.6	M
LJAMM-CNP 7670	55	22	11	11.3	?	10.5	8.6	5.8	4.2	4.9	4.6	4.5	1.8	F
LJAMM-CNP 7674	50	23	9	9.9	11.9	11.8	8.3	6.3	4.1	4.8	4.7	4.5	1.6	F
LJAMM-CNP 7804	52	20	11	10.2	12.1	9.8	10.1	6.1	2.9	3.6	4.7	5.3	2.3	F
LJAMM-CNP 8713	56	25	8	10.5	?	?	10.6	?	?	?	?	4.8	2.2	M

Specimen	SVL	TrL	FL	TL	AL	HL	HW	HH	END	ESD	EMD	ID	IND	Sex
LJAMM-CNP 10493	53	21	10	10.4	14.4	11.7	8.7	?	3.7	4.6	4.5	4.5	2.0	M
LJAMM-CNP 10496	51	23	9	9.4	12.9	12.5	9.9	5.5	4.0	4.8	5.1	4.4	1.8	F
LJAMM-CNP 10523	57	22	?	10.3	14.5	14.5	12.4	6.7	4.8	6.0	5.9	5.8	1.8	F
LJAMM-CNP 10526	64	27	12	12.0	16.8	14.3	10.8	6.3	4.9	6.1	5.5	5.2	1.9	F
LJAMM-CNP 10576	51	18	9	10.1	12.7	13.2	10.8	6.1	4.5	5.8	5.3	4.6	1.8	M
LJAMM-CNP 10577	42	16	8	8.3	11.8	10.6	8.5	4.9	3.7	4.2	4.2	4.1	1.2	F
LJAMM-CNP 10578	47	17	9	9.5	14.2	14.1	9.2	6.7	4.8	6.0	5.5	4.4	2.2	F
LJAMM-CNP 10579	53	20	?	10.4	13.1	13.4	9.9	6.4	4.1	5.3	5.3	4.1	2.0	F
LJAMM-CNP 10584	59	23	9	11.4	14.7	16.1	10.4	7.8	5.0	6.6	6.5	5.1	2.3	M
LJAMM-CNP 13948	44	19	9	8.9	12.8	10.7	8.2	5.5	3.2	4.3	4.4	4.1	1.7	M
LJAMM-CNP 14551	51	24	9	9.9	12.8	12.0	10.0	?	3.9	5.0	4.7	4.8	1.5	M
MNHNP 2821	61	28	12	9.9	14.6	14.4	11	7.7	4.5	5.9	5.3	4.8	2.4	F
MNHNP 9037	65	28	12	11.3	15	17.3	12.4	8.6	5.8	6.8	6.0	5.2	2.5	F
MNHNP 9038	51	23	9	9.8	13.1	13.1	10.2	7.1	4.3	5.1	4.9	4.4	2	M
MNHNP 9131	41	20	9	8.4	11.8	11.4	9.5	6.4	3.9	3.6	4.4	4.2	1.4	M
MNHNP 11406	47	18	7	8.3	11.5	11.4	8.8	6.3	3.7	5	3.6	4.1	1.8	?

Specimen	SVL	TrL	FL	TL	AL	HL	HW	HH	END	ESD	EMD	ID	IND	Sex
MNHNP 11410	39	15	8	7.2	10.2	10.7	8.1	5.8	3.7	4.4	3.6	3.7	1.7	M
MNHNP 11419	40	17	8	7.6	9.4	10.5	8.1	6.6	3.4	4.3	3.6	3.5	1.5	?
MNHNP 11421	45	17	9	8.1	11	10.9	9.2	6.7	3.7	5	3.9	3.8	1.7	M
MNHNP 11423	45	19	9	9.1	11.6	11.6	9	6.4	3.4	4.5	4.0	4	1.6	M
MNHNP 11850	53	25	9	9.8	12.2	13.3	10.6	7.9	4.7	5.9	4.5	5.1	2.5	M
MNHNP 11855	58	28	11	11.1	13.6	13.7	11.2	8.4	4.6	6.4	5.6	5.3	2.3	F
MNHNP 11860	51	23	10	9.4	13.1	12.9	9.7	6.8	4.9	5.8	4.6	4.6	1.9	F
MNHNP 11872	48	20	9	8.4	11.8	11.5	8.9	6.4	4.5	4.9	3.8	3.8	1.9	M
MNHNP 12238	60	26	11	10.8	14.1	14.8	13.3	7.9	4.6	6.6	5.1	5.5	2.5	F
SMF 29277	37	16	6	7.1	9.9	9.8	7.8	6.4	2.8	4.2	3.6	3.6	1.9	?
SMF 101984	53	24	9	9.8	13.2	13.4	10.4	6.7	4.1	5.2	4.6	4.6	1.9	?

Appendix S12

Matrix of meristic data

Raw meristic data used for analyses. See Materials and Methods section for explanation of the characters. Missing data indicated with "?". Argentinean populations belong to LJAMM-CNP collection, whereas Paraguayan specimens are housed in the MNHNP and SMF.

Specimen	DT	TVS	LVS	SL	IF	4TL	3FL	Sex
LJAMM-CNP 6520	30	43	19	9	7	19	16	F
LJAMM-CNP 6530	28	45	15	9	7	20	14	F
LJAMM-CNP 6532	33	48	16	7	6	18	13	M
LJAMM-CNP 6533	31	36	15	8	7	19	14	F
LJAMM-CNP 6535	34	44	18	8	7	21	17	M
LJAMM-CNP 6967	34	42	17	9	7	16	15	M
LJAMM-CNP 6968	30	42	18	8	8	17	15	M
LJAMM-CNP 7670	35	47	14	8	6	20	14	F
LJAMM-CNP 7674	35	40	13	8	6	20	14	F
LJAMM-CNP 7804	34	43	17	8	7	20	16	F
LJAMM-CNP 8713	32	48	17	8	6	20	16	M

Specimen	DT	TVS	LVS	SL	IF	4TL	3FL	Sex
LJAMM-CNP 10493	32	39	14	8	6	17	14	M
LJAMM-CNP 10496	33	39	16	8	6	18	15	F
LJAMM-CNP 10523	32	41	15	8	6	19	14	F
LJAMM-CNP 10526	34	39	13	8	6	21	16	F
LJAMM-CNP 10576	33	46	20	8	7	22	17	M
LJAMM-CNP 10577	34	42	16	7	7	20	16	F
LJAMM-CNP 10578	36	42	19	9	7	21	15	F
LJAMM-CNP 10579	37	42	15	8	7	?	16	F
LJAMM-CNP 10584	33	43	16	9	6	20	15	M
LJAMM-CNP 13948	29	42	14	8	6	18	14	M
LJAMM-CNP 14551	36	45	16	9	8	21	14	M
MNHNP 2821	29	42	17	8	6	20	14	F
MNHNP 9037	34	42	16	7	6	19	15	F
MNHNP 9038	31	40	12	7	6	17	12	M
MNHNP 9131	29	40	17	8	6	17	13	M
MNHNP 11406	32	?	?	8	6	19	13	?

Specimen	DT	TVS	LVS	SL	IF	4TL	3FL	Sex
MNHNP 11410	28	?	?	9	6	?	13	M
MNHNP 11419	29	43	15	8	6	18	11	?
MNHNP 11421	28	41	15	9	7	17	13	M
MNHNP 11423	32	41	19	8	6	19	14	M
MNHNP 11850	31	39	18	8	6	18	12	M
MNHNP 11855	28	43	17	8	6	16	12	F
MNHNP 11860	31	37	19	6	7	18	12	F
MNHNP 11872	32	47	16	8	7	18	13	M
MNHNP 12238	32	42	19	9	6	17	13	F
SMF 29277	34	42	15	8	6	17	11	?
SMF 101984	33	39	17	7	7	20	13	?
